# Minimal Dispersion on the Sphere

**DOI:** 10.1007/s00454-025-00812-8

**Published:** 2026-08-20

**Authors:** Alexander E. Litvak, Mathias Sonnleitner, Tomasz Szczepanski

**Affiliations:** 1 Department of Mathematical and Statistical Sciences, University of Alberta, Edmonton, T6G 2G1 AB Canada; 2 Present Address: Institute of Financial Mathematics and Applied Number Theory, Johannes Kepler University Linz, Altenbergerstrasse 69, Linz, 4040 Austria

**Keywords:** Dispersion, Covering density, Covering radius, Largest empty cap, Primary: 52B55, 52C17, Secondary: 52A23, 52C45

## Abstract

The minimal spherical cap dispersion ${{\,\textrm{disp}\,}}_{\mathcal {C}}(n,d)$ is the largest number $\varepsilon \in (0,1]$ such that, for every *n* points on the *d*-dimensional Euclidean unit sphere $\mathbb {S}^d$, there exists a spherical cap with normalized area ε not containing any of these points. We study the behavior of ${{\,\textrm{disp}\,}}_{\mathcal {C}}(n,d)$ as *n* and *d* grow to infinity. We develop connections to the problems of sphere covering and approximation of the Euclidean unit ball by inscribed polytopes. Existing and new results are presented in a unified way. Upper bounds on ${{\,\textrm{disp}\,}}_{\mathcal {C}}(n,d)$ result from choosing the points independently and uniformly at random and possibly adding some well-separated points to close large gaps. Moreover, we study dispersion with respect to intersections of caps.

## Introduction and results

Given a point set $P=\{x_1,\dots ,x_n\}$ on the *d*-dimensional Euclidean unit sphere $\mathbb {S}^d\subset \mathbb {R}^{d+1}$, where d≥1, define its spherical cap dispersion by

$$ {{\,\textrm{disp}\,}}_{\mathcal {C}}(P) :=\sup _{C\in \mathcal {C}}\{\sigma (C):C\cap \{x_1,\dots ,x_n\}=\emptyset \}, $$

where $\mathcal {C}$ denotes the family of spherical caps of $\mathbb {S}^d$ and σ is the normalized Lebesgue measure on $\mathbb {S}^d$, which we also call the spherical measure. We define the minimal spherical cap dispersion by

1.1 $$\begin{aligned} {{\,\textrm{disp}\,}}_{\mathcal {C}}(n,d) :=\inf _{x_1,\dots ,x_n\in \mathbb {S}^{d}} {{\,\textrm{disp}\,}}_{\mathcal {C}}(\{x_1,\dots ,x_n\}). \end{aligned}$$

We are interested in the asymptotic behavior of ${{\,\textrm{disp}\,}}_{\mathcal {C}}(n,d)$ as *n* and *d* grow to infinity.

In the case of the unit cube $[0,1]^d$ equipped with the Lebesgue measure and the family of axis-parallel boxes, the minimal dispersion was introduced in the context of uniform distribution theory by Hlawka [40] and Niederreiter [50]. We refer to [2, 15, 45, 46, 62, 63] and references therein for history and recent progress.

In the case of $\mathbb {S}^d$, the minimal dispersion has been studied for spherical slices (intersections of half-spheres) by Rote and Tichy [56] and subsequently by Prochno and Rudolf [51] for intersections of two spherical caps, that is for the family

$$ \mathcal {L}=\{C_1\cap C_2:C_1,C_2\in \mathcal {C}\}, $$

which we refer to as spherical lenses. The corresponding notion of minimal lens dispersion is then given by

1.2 $$\begin{aligned} {{\,\textrm{disp}\,}}_{\mathcal {L}}(n,d) :=\inf _{x_1,\dots ,x_n\in \mathbb {S}^{d}} \,\, \sup _{A\in \mathcal {L}}\, \{\sigma (A):A\cap \{x_1,\dots ,x_n\}=\emptyset \}. \end{aligned}$$

The study of spherical dispersion is motivated by a question of Erdős [25, p. 54] who asked for a lower bound on the minimal spherical cap discrepancy

$$ D(n,d) :=\inf _{x_1,\dots ,x_n\in \mathbb {S}^d}\sup _{C\in \mathcal {C}} \Big | \frac{|\{i:x_i\in C\}|}{n} - \sigma (C)\Big |, $$

where the supremum is taken over the system of spherical caps $\mathcal {C}$ (see Blümlinger [10] for the spherical slice discrepancy). A lower bound for *D*(*n*, *d*) was given by Schmidt [58] and improved by Beck [6], who had provided an almost matching upper bound in [5]. It is a well-known open problem to determine the asymptotic behavior of *D*(*n*, *d*) as $n\rightarrow \infty$ for d≥2. Clearly, $D(n,d)\ge {{\,\textrm{disp}\,}}_{\mathcal {C}}(n,d)$ and, as we present below, the behavior of ${{\,\textrm{disp}\,}}_{\mathcal {C}}(n,d)$ is better understood than that of *D*(*n*, *d*). At the end of the introduction we will comment on ${{\,\textrm{disp}\,}}_{\mathcal {L}}(n,d)$.

Recall that a family $\mathcal {C}$ of spherical caps coincides with the family of geodesic balls

$$ B(x,\varphi )=\{y\in \mathbb {S}^d:\varrho (x,y)\le \varphi \},\qquad x\in \mathbb {S}^d,\varphi \in [0,\pi ], $$

where $\varrho (x,y)=\arccos (\langle x,y\rangle )$ denotes the geodesic distance. Note that the dispersion remains unchanged whether we consider open or closed caps, and we work with the latter. We start with the observation that the minimal spherical cap dispersion is related to covering the sphere by caps. Define the minimal spherical covering density of the sphere by

$$ {{\,\textrm{dens}\,}}(n,d) := \inf \Big \{\sum _{i=1}^{n}\sigma (B(x_i,\varphi )):\bigcup _{i=1}^n B(x_i,\varphi )= \mathbb {S}^d, x_1,\dots ,x_n\in \mathbb {S}^d, \varphi \in (0,\pi ]\Big \} $$

and the minimal geodesic covering radius by

$$ \varphi (n) :=\inf \Big \{\varphi >0:\exists x_1,\dots ,x_n\in \mathbb {S}^d \text { with }\bigcup _{i=1}^n B(x_i,\varphi )=\mathbb {S}^d\Big \}. $$

These two parameters have been intensively studied, see the books and surveys [3, 11, 20, 27, 29, 30, 55]. We summarize the relations between them and dispersion in the following lemma, which will be proved in Section 2.2.

### Lemma 1.1

Let *d*, *n* be positive integers. Then

1.3 $$\begin{aligned} n\cdot {{\,\textrm{disp}\,}}_{\mathcal {C}}(n,d) ={{\,\textrm{dens}\,}}(n,d) =n\cdot V(\varphi (n)), \end{aligned}$$

where $V(\varphi )=\sigma (B(x,\varphi ))$ denotes the normalized volume of a cap with geodesic radius $\varphi \in [0,\pi ]$ centered at $x\in \mathbb {S}^d$.

In the following, we derive statements about behavior of the spherical cap dispersion. It is easy to see that ${{\,\textrm{disp}\,}}_{\mathcal {C}}(n,d)\ge \frac{1}{n}$ (which also follows from ${{\,\textrm{dens}\,}}(n,d)\ge 1$ and (1.3)). When n≤d+1 the affine hull of *n* points spans an affine subspace of $\mathbb {R}^{d+1}$ with dimension at most *d*. Moreover, any cap of area at least $\frac{1}{2}$ intersects a given pair of antipodal points. Therefore we have

1.4 $$\begin{aligned} {{\,\textrm{disp}\,}}_{\mathcal {C}}(1,d)=1 \quad \quad \text{ and } \quad \quad {{\,\textrm{disp}\,}}_{\mathcal {C}}(2,d)=\cdots ={{\,\textrm{disp}\,}}_{\mathcal {C}}(d+1,d)=\frac{1}{2}. \end{aligned}$$

For $d+2\le n\le 2d+2$ optimizers are expected to be regular structures, see [12, Conjecture 1.3] and [11, Chapter 6]. More precisely, for n=d+1+k, $1\le k\le d+1$, split $\mathbb {R}^{d+1}$ into an orthogonal sum of *k* subspaces $E_i$ of the dimensions $\lceil (d+1)/k\rceil$ and $\lfloor (d+1)/k\rfloor$ and in each $E_i$ take a regular simplex inscribed into the Euclidean ball. Then take convex hull of such simplices. In particular, for n=d+2 (so k=1) a regular simplex inscribed into $\mathbb {S}^d$ gives the optimal covering with radius $\varphi (d+2)=\arccos \frac{1}{d+1}$ and for n=2d+2 (so k=d+1) the cross-polytope inscribed into $\mathbb {S}^d$ yields a covering with *n* caps of radius $\arccos \frac{1}{\sqrt{d+1}}$, which is conjectured to be optimal (see also [22, Problem 4]). We note also that such arrangements were used to prove sharpness of the lower bound on the distance between convex polytopes with few vertices and centrally-symmetric bodies [31]. It follows from volume bounds given in Section 2.1 (see Remark 2.3) that

1.5 $$\begin{aligned} \frac{1}{2}-{{\,\textrm{disp}\,}}_{\mathcal {C}}(d+2,d)\sim \frac{1}{\sqrt{2\pi d}}, \end{aligned}$$

where for sequences $(a_d)_d$ and $(b_d)_d$ of positive numbers we write $a_d\sim b_d$ for $\lim _{d\rightarrow \infty }\frac{a_d}{b_d}=1$. Moreover, if n=2d+2, then

1.6 $$\begin{aligned} {{\,\textrm{disp}\,}}_{\mathcal {C}}(2d+2,d)\le \frac{1+o(1)}{\sqrt{2\pi }}\int _1^{\infty }e^{-x^2/2}\,\textrm{d}x \qquad \text {as }d\rightarrow \infty \end{aligned}$$

(see Lemma 2.4 and Remark 2.5 below).

### Remark 1.2

For completeness we remark that in the case of d=1 the sphere $\mathbb {S}^1$ is a one-dimensional torus and ${{\,\textrm{disp}\,}}_{\mathcal {C}}(n,1)=\frac{1}{n}$ for every $n\in \mathbb {N}$. Thus, we focus on d≥2. We refer to [2, 45, 46, 57, 64] about dispersion with respect to periodic axis-parallel boxes on a torus of dimension d≥2.

If *n* is sufficiently large compared to *d*, then local approximation of $\mathbb {S}^d$ by $\mathbb {R}^d$ suggests that ${{\,\textrm{dens}\,}}(n,d)$ is related to the minimal covering density of $\mathbb {R}^d$ by equally sized balls. The latter can be defined by

1.7 $$\begin{aligned} \vartheta _d =\inf _{\mathcal {B}}\lim _{R\rightarrow \infty }\sum _{B\in \mathcal {B}} \frac{{{\,\textrm{vol}\,}}_d(B\cap B^d(0,R))}{{{\,\textrm{vol}\,}}_d(B^d(0,R))}, \end{aligned}$$

where the infimum is taken over any covering $\mathcal {B}=\{B^d(x_1,1),B^d(x_2,1),\dots \}$ of $\mathbb {R}^d$ by Euclidean balls of unit radius. Indeed, in the next theorem we show that $\lim _{n\rightarrow \infty }{{\,\textrm{dens}\,}}(n,d)=\vartheta _d$ (we state all our results in the equivalent notion of dispersion, see Lemma 1.1).

### Theorem 1.3

Let d≥2. Then the minimal dispersion satisfies

$$ \lim _{n\rightarrow \infty }n\cdot {{\,\textrm{disp}\,}}_{\mathcal {C}}(n,d) =\vartheta _d. $$

Instead of proving Theorem 1.3 directly, we deduce it from the known asymptotic for the best approximation of the Euclidean unit ball $B^{d+1}$ by inscribed polytopes with *n* vertices in Hausdorff distance (see e.g., [59]).

The minimal covering density of $\mathbb {R}^d$ is known to satisfy $\vartheta _2=\frac{2\pi }{3\sqrt{3}}$ in the case d=2 which is attained by the hexagonal lattice (see [43]) and, for d≥3,

1.8 $$\begin{aligned} \frac{d}{e\sqrt{e}}\sim \tau _d \le \vartheta _d \le d\ln d+d\ln \ln d+5d. \end{aligned}$$

The lower bound in (1.8) is due to Coxeter-Few-Rogers [21] and the upper is due to Rogers [53], which was improved by Fejes Tóth [28] by replacing the summand 5*d* with *d* and by Dumer [24] who obtained the bound (1/2+o(1))dlnd. The constant $\tau _d$ can be written explicitly in terms of the interior angle of a *d*-dimensional regular simplex.

### Remark 1.4

The upper bound in (1.8) also holds for coverings by other convex shapes, while no nontrivial lower bound for shapes other than the ball is known so far, see Fejes Tóth [35, p. 36]. We refer to [16] for recent results developments in this direction.

It follows from Theorem 1.3 and the bounds in (1.8) that

1.9 $$\begin{aligned} d\lesssim \lim _{n\rightarrow \infty }n\cdot {{\,\textrm{disp}\,}}_{\mathcal {C}}(n,d)\lesssim d\ln d, \end{aligned}$$

where for sequences $(a_d)_d$ and $(b_d)_d$ of positive numbers we write $a_d\lesssim b_d$ if for some C>0 and all *d* one has $a_d \le C\,b_d$. Note that compared to the minimal dispersion on the cube with respect to axis-parallel boxes (see Bukh and Chao [15], who showed *cd* for the lower bound and $Cd^2 \ln d$ for the upper bound) we have only a logarithmic gap between the lower and upper bounds in the case of the spherical cap dispersion.

Regarding the upper bound in (1.8), Erdős and Rogers [26] have shown that there exists a covering of $\mathbb {R}^d$ by unit balls such that no point is covered by more than $e(d\ln d + d\ln \ln d+5d)$ balls. In the case of the sphere, Böröczky Jr. and Wintsche [12] proved that for any $\varphi <\frac{\pi }{2}$ there is a covering of $\mathbb {S}^d$ by caps of geodesic radius φ such that no point of $\mathbb {S}^d$ is covered by more than 400dlnd caps. From this one can derive the following bound.

### Proposition 1.5

Let d,n≥2. Then the minimal dispersion satisfies

$$\begin{aligned} n\cdot {{\,\textrm{disp}\,}}_{\mathcal {C}}(n,d)\le 400 d\ln d. \end{aligned}$$

In fact, in [12] (Remark 5.1 and the end of its proof, see also [48, Theorem 2.2]) the authors proved also that there is a covering of the sphere with the density bounded by $d\ln d+d\ln \ln d+5d$ (note, this is the same bound as in (1.8)). This leads to the following estimate.

### Proposition 1.6

Let d,n≥2. Then the minimal dispersion satisfies

$$ n\cdot {{\,\textrm{disp}\,}}_{\mathcal {C}}(n,d) \le d\ln d+d\ln \ln d+5d. $$

The bound in Proposition 1.6 can be further improved as follows. Corollary 1.2 in [12] states that for every $\varphi \in (0,\arccos \frac{1}{\sqrt{d+1}})$ there is a covering of $\mathbb {S}^d$ by at most

1.10 $$\begin{aligned} C\,\frac{\cos \varphi }{\sin ^d\varphi }\cdot d^{3/2}\ln (1+d\cos ^2\varphi ) \end{aligned}$$

geodesic balls of radius φ with density bounded by $c \ln (1+d\cos ^2\varphi )$, where C,c>0 are absolute constants. From this we deduce the following bound which improves Proposition 1.6 if *n* is subexponential in *d*.

### Theorem 1.7

Let d≥2. Then the minimal dispersion satisfies

$$ n\cdot {{\,\textrm{disp}\,}}_{\mathcal {C}}(n,d)\le C\, d\ln \ln \frac{2n}{d}\qquad \text {for }\,\,\, n\ge 2d, $$

where C>1 is an absolute constant.

In particular, Theorem 1.7 shows that ${{\,\textrm{disp}\,}}_{\mathcal {C}}(cd,d)$ can be made arbitrarily small by choosing *c* large enough. The same is true for the spherical cap discrepancy (see [38, Theorem 3] which may be adapted to our situation using Lemma 5.2 below). This is in contrast to the related problem of approximation of the Euclidean ball $B^{d+1}$ by inscribed polytopes with *n* vertices, where *n* needs to grow faster than $d^{d/2}$ for the error to converge to zero, which follows from bounds provided in [4, 18, 32] (which are essentially inequality (2.7) below).

Theorem 1.7 and Lemma 1.1 imply that $V(\varphi (n))\le C\, (d/n) \ln \ln (2n/d)$. This immediately provides a bound on covering numbers of the sphere. Recall that a set of points $\mathcal {N}\subset \mathbb {S}^d$ is called a (geodesic) ε-net if for every $x\in \mathbb {S}^d$ there is $y\in \mathcal {N}$ such that $\varrho (x,y)\le \varepsilon$. Therefore, adjusting the constant *C* we have the following bound.

### Corollary 1.8

For every d≥2 and $\varepsilon \in (0, \pi /2)$ there exists a geodesic ε-net of $\mathbb {S}^d$ with cardinality bounded by

$$ d\, \max \Big \{2, \, \frac{C\,\ln \ln (2/V(\varepsilon ))}{V(\varepsilon )}\Big \}, $$

where C>0 is an absolute constant.

This estimate complements standard bounds (see for example Corollary 5.5 in [3]) and should be compared with bounds in Examples 6.1 and 6.2 in [12].

Moreover, the results of [12] imply the following lower bound, which unfortunately holds only for *n* superexponential in *d*. This restriction appears as the proof uses the lower bound in (1.8) for $\mathbb {R}^d$ and a sufficiently good approximation of $B^{d+1}$ by a polytope with *n* vertices. We would like to note that a similar restriction on *n* also appears (although for a different reason) in the lower bound in [15] for the minimal dispersion in the case of the cube.

### Theorem 1.9

Let d≥2. Then the minimal dispersion satisfies

$$ n\cdot {{\,\textrm{disp}\,}}_{\mathcal {C}}(n,d) \ge c_0\,d \qquad \text {for }\,\,\, n\ge C d^{(d+3)/2}\ln d, $$

where $c_0, C>0$ are absolute constants.

Note that, asymptotically as $n\rightarrow \infty$, the constant $c_0$ in Theorem 1.9 can be chosen as $\frac{1}{e\sqrt{e}}$ by Theorem 1.3.

Several upper bounds, including the ones in Propositions 1.5, 1.6 and Theorem 1.7 (all derived from [12]), rely on semi-random constructions of identically and independently distributed (i.i.d.) random points (with respect to the probability measure σ) with well-separated points to fill the gaps left by the random points. Such a technique goes back at least to Rogers [53], who used it for the upper bound of (1.8) and for the spherical covering density in [54]. The book [3, Chapter 5] contains a presentation of this technique for the case of covering the sphere by spherical caps. It was also recently used in [2] for the minimal dispersion on the cube. Note that Naszódi [48] used non-probabilistic tools to reprove the bound from [12] mentioned before Proposition 1.6.

If the number of points *n* is large compared to the dimension *d*, then covering of $\mathbb {S}^d$ relying only on i.i.d. random centers may introduce an additional logarithmic factor in *n*. This is because the largest gap between i.i.d. random points is logarithmically larger than the average gap, see [17, 42, 52] for details (note that in the case d=1 it is an easy fact). This effect is related to the coupon collector’s problem (see [39] for dispersion on the cube).

### Remark 1.10

In a recent work, Hoehner and Kur [41] have shown that covering most of the sphere using i.i.d. random points is asymptotically optimal in high dimension. More precisely, the maximum proportion of the sphere $\mathbb {S}^d$ covered by *n* spherical caps of volume $\frac{1}{n}$ tends to $1-\frac{1}{e}$ as *n* and *d* tend to infinity.

A separate question is what bounds can be obtained by using a random choice of points (with respect to the normalized Lebesgue measure). Such a choice of points was used in recent works on dispersion of the cube [45, 46, 57] and for spherical lens dispersion [51]. Moreover, the above mentioned bounds in [57] and [51] are immediate consequences of a result in [9], which essentially uses the so-called ε-net theorem in combinatorics due to [37] (see Theorem 5.1 below). We would like to note that this ε-net theorem has been used by Naszódi [49] to construct a good approximation of a convex body by convex polytopes with small number of vertices. Below, we use the results from [9, 37] to derive the following bound, which is slightly weaker than the one in Theorem 1.7 but relies only on i.i.d. points.

### Proposition 1.11

Let d≥2. Then the minimal dispersion satisfies

$$ n\cdot {{\,\textrm{disp}\,}}_{\mathcal {C}}(n,d) \le \frac{3(d+2)}{\ln 2}\ln \Big (\frac{2e n}{d+2}\Big ) \qquad \text {for }\,\,\, n\ge d+2. $$

Moreover, the bound holds for independent uniformly distributed points on $\mathbb {S}^d$.

In fact, it can be deduced from the proof that the bound holds with probability 1-α, $\alpha \in (0,1)$, for i.i.d. random points if the factor 3 is replaced by a large enough constant depending only on α. Although the proof of the combinatorial ε-net theorem is not very long (for the sake of completeness, we present it in Section 5 together with the proof of Proposition 1.11), there is an even simpler approach using a standard (geometric) ε-net argument, which however yields a slightly worse bound in the regime $d+2\le n \le Cd^2$ than Proposition 1.11.

### Proposition 1.12

Let d,n≥2. Then the minimal dispersion satisfies

$$ n\cdot {{\,\textrm{disp}\,}}_{\mathcal {C}}(n,d) \le 12d\ln n. $$

Moreover, the bound holds for independent uniformly distributed points on $\mathbb {S}^d$.

The proof of Proposition 1.12 will be given in Section 4 and it is based on the idea of δ-approximations which were discussed in [46] (see also [2, 45]) for the dispersion on the cube as a simplification of the notion of δ-covers used in [23] on the sphere. In the preprint [13], a predecessor of [51], an approach using δ-covers was carried out, giving bounds of the same order as in Proposition 1.12. We refer to [33, 34, 57] for δ-covers and related notions on the cube. Let us note that δ-covers were originally used in discrepancy theory from which the concept of dispersion emerged.

The minimal lens dispersion, i.e. ${{\,\textrm{disp}\,}}_{\mathcal {L}}(n,d)$ defined in (1.2), is more complicated to analyze since there is no direct connection to covering. Theorem B in [51] asserts that

1.11 $$\begin{aligned} n\cdot {{\,\textrm{disp}\,}}_{\mathcal {L}}(n,d) \le C d\ln \Big (\frac{en}{32d}\Big )\qquad for \,\,\, n\ge 32d, \end{aligned}$$

where C>0 is an absolute constant. Moreover, this holds for the expected dispersion of *n* i.i.d. random points and the proof is based on bounds derived from [9] (analogous to our Theorem 5.1). For completeness, we provide a proof of Theorem 5.1 and inequality (1.11) in Section 5. In [13] a suboptimal bound using δ-covers was given. We recover this bound with a simpler proof building on δ-approximations as used for Proposition 1.12.

### Proposition 1.13

Let d,n≥2. Then the minimal lens dispersion satisfies

$$ n\cdot {{\,\textrm{disp}\,}}_{\mathcal {L}}(n,d) \le 24(d+1)\ln n . $$

Moreover, the bound holds for independent uniformly distributed points on $\mathbb {S}^d$.

We finally would like to note that the bound (1.11) and Proposition 1.13 extend to families generated by intersections of an arbitrary number of caps with essentially the same proof. For future reference we formulate it below and prove it in the last section.

### Theorem 1.14

Let d,k≥2 and

$$ \mathcal {L}_k =\{C_1\cap C_2\cap ...\cap C_k \, :\, C_1, C_2, ..., C_k \in \mathcal {C}\}. $$

Let ${{\,\textrm{disp}\,}}_{\mathcal {L}_k}(n,d)$ be defined similarly to ${{\,\textrm{disp}\,}}_{\mathcal {C}}(n,d)$ and ${{\,\textrm{disp}\,}}_{\mathcal {L}}(n,d)$. Then

$$ n\cdot {{\,\textrm{disp}\,}}_{\mathcal {L}_k}(n,d) \le C d\, k \ln k \, \ln \Big (\frac{n}{c d\, k \ln k}\Big )\qquad for \,\,\, n\ge C \,d\, k \ln k, $$

where c,C>0 are absolute constants.

The structure of this note is as follows. In Section 2.1 we review volume bounds for spherical caps and in Lemma 2.6 we provide a bound for the cardinality of a geodesic ε-net on $\mathbb {S}^d$. Section 2.2 is devoted to the proof of Lemma 1.1. In Section 2.3 we present the relation between dispersion and approximation of the ball by polytopes. The subsequent sections contain proofs of theorems and propositions. Section 4 contains the proofs of Proposition 1.12 and Proposition 1.13 using standard (geodesic) ε-nets and in Section 5 we present proofs of dispersion bounds (Proposition 1.11, inequality (1.11), and Theorem 1.14) using VC-dimension.

## Preliminaries

### Volume Bounds

The normalized volume of a spherical cap of geodesic radius $\varphi \in [0,\pi ]$ can be computed by

2.1 $$\begin{aligned} V(\varphi ) =\frac{{{\,\textrm{vol}\,}}_{d-1}(\mathbb {S}^{d-1})}{{{\,\textrm{vol}\,}}_d(\mathbb {S}^d)}\int _0^{\varphi }\sin ^{d-1}t\,\textrm{d}t = \frac{\int _0^\varphi \sin ^{d-1}t\,\textrm{d}t}{2\int _0^\frac{\pi }{2} \sin ^{d-1}t\,\textrm{d}t}, \end{aligned}$$

where the *d*-dimensional volume of $\mathbb {S}^d$ is given by

$$\begin{aligned} {{\,\textrm{vol}\,}}_d(\mathbb {S}^d) =\frac{2\pi ^{(d+1)/2}}{\Gamma (\frac{d+1}{2})}. \end{aligned}$$

It is known that the Wallis integral $\int _0^\frac{\pi }{2} \sin ^{d-1}t\,\textrm{d}t$ satisfies

2.2 $$\begin{aligned} \sqrt{\frac{\pi }{2d}} \le \int _0^{\frac{\pi }{2}}\sin ^{d-1}t\,\,\textrm{d}t \le \sqrt{\frac{\pi }{2(d-1)}}\qquad \text {for}\,\,\, d\ge 2, \end{aligned}$$

(which follows from [7, Lemma 1]). We need the following volume bounds.

#### Lemma 2.1

Let d≥2.

(i) If $$\varphi \in (0,\frac{\pi }{2})$$, then
  $$ \frac{1}{\sqrt{2\pi (d+1)}}\sin ^d\varphi \le V(\varphi ) \le \frac{1}{2}\sin ^d\varphi . $$
(ii) If $\varphi \le \arccos \frac{1}{\sqrt{d+1}}$, then
  $$ \frac{1}{3\sqrt{2\pi (d+1)}}\cdot \frac{1}{\cos \varphi } \sin ^d\varphi \le V(\varphi ) \le \frac{1}{\sqrt{2\pi d}}\cdot \frac{1}{\cos \varphi } \sin ^d\varphi . $$
(iii) If $\varphi \ge \arccos \frac{1}{\sqrt{d+1}}$, then
  $$ \frac{1}{3e\sqrt{2\pi }} \le V(\varphi ) \le \frac{1}{2}. $$
(iv) If $\alpha \in (0,\frac{\pi }{2})$, then
  $$ e^{-\frac{(d-1) \alpha ^2}{2\cos ^2 \alpha }}\sqrt{\frac{d-1}{2\pi }}\,\alpha \le \frac{1}{2}-V\Big (\frac{\pi }{2}-\alpha \Big ) \le \sqrt{\frac{d}{2\pi }}\,\alpha . $$
(v) If $$0<\delta \le w \le \pi $$, then
  $$ \frac{V(w)}{V(\delta )}\le \Big (\frac{w}{\delta }\Big )^d. $$

#### Proof

The upper bound in (i) is taken from [3, Proposition 5.1]. The lower bound in (i) and bounds in (ii) and (iii) are taken from [12, Corollary 3.2].

To prove (iv) write

2.3 $$\begin{aligned} \frac{1}{2}-V\Big (\frac{\pi }{2}-\alpha \Big )= &   \frac{1}{2}-\frac{\int _0^{\frac{\pi }{2}-\alpha }\sin ^{d-1}t\,\,\textrm{d}t}{2\int _0^{\frac{\pi }{2}}\sin ^{d-1}t\,\,\textrm{d}t}\nonumber \\= &   \frac{\int _{\frac{\pi }{2}-\alpha }^{\frac{\pi }{2}+\alpha }\sin ^{d-1}t\,\,\textrm{d}t}{4\int _0^{\frac{\pi }{2}}\sin ^{d-1}t\,\,\textrm{d}t}. \end{aligned}$$

The numerator in (2.3) can be written as

$$ \int _{\frac{\pi }{2}-\alpha }^{\frac{\pi }{2}+\alpha }\sin ^{d-1}t\,\,\textrm{d}t = \int _{-\alpha }^{\alpha }\cos ^{d-1}t\,\,\textrm{d}t = \int _{-\alpha }^{\alpha }e^{(d-1)\, f(t)}\,\textrm{d}t, $$

where $$f(t)=\ln \cos t$$ on $$(-\frac{\pi }{2},\frac{\pi }{2})$$ satisfies f(0)=0, $f^{\prime }\left(0\right)=0$ and $$f''(t)=-\frac{1}{\cos ^2 t}$$. Consequently,

$$ -\frac{1}{\cos ^2 \alpha }\frac{t^2}{2} \le f(t) \le -\frac{t^2}{2}\qquad \text {for }t\in (-\alpha ,\alpha ). $$

We bound the numerator in (2.3) from above by

$$ \int _{-\alpha }^{\alpha }e^{(d-1)\,f(t)}\,\textrm{d}t \le \int _{-\alpha }^{\alpha }e^{-(d-1)\frac{t^2}{2}}\,\textrm{d}t \le 2\alpha , $$

and from below by

$$ \int _{-\alpha }^{\alpha }e^{(d-1)\,f(t)}\,\textrm{d}t \ge \int _{-\alpha }^{\alpha }e^{-\frac{d-1}{\cos ^2 \alpha }\frac{t^2}{2}}\,\textrm{d}t \ge 2\alpha e^{-\frac{(d-1)\alpha ^2}{2\cos ^2 \alpha }}. $$

Combined with (2.2) this completes the proof of (iv).

The bound in (v) follows from the Bishop-Gromov volume comparison theorem (see e.g. [19, Theorem III.4.5]). Note that it is essentially proven using elementary tools in [12, Lemma 3.1 (ii)] for $0<\delta \le w\le \pi /2$. For convenience of the reader we extend the proof to $0<\delta \le w\le \pi$. We will use the following fact.

#### Claim 2.2

Let d≥2, $0< \delta \le \pi$. Then for every $1\le t\le \frac{\pi }{\delta }$,

(i) $$\sin (t\delta ) \le t\sin (\delta ),$$
(ii) $$\delta \sin ^{d-1}\delta \le d\int _0^\delta \sin ^{d-1} s\,\textrm{d}s.$$

#### Proof

Note that the function $\frac{\sin x}{x}$ is decreasing on (0,π]. Since $1\le t\le \frac{\pi }{\delta }$, this implies that

$$\begin{aligned} \frac{\sin (t\delta )}{t\sin \delta } = \frac{\sin (t\delta )}{t\delta }\cdot \frac{\delta }{\sin \delta } \le 1, \end{aligned}$$

which proves (i).

To prove (ii) consider the function $f(\delta ) = d\int _0^\delta \sin ^{d-1} s\,\textrm{d}s - \delta \sin ^{d-1}\delta$ on (0,π]. Then f(0)=0 and

$$\begin{aligned} f'(\delta )&= d\sin ^{d-1}\delta - \sin ^{d-1}\delta - (d-1)\delta \sin ^{d-2}\delta \cos \delta \\&= (d-1)\sin ^{d-2}\delta (\sin \delta - \delta \cos \delta ). \end{aligned}$$

Since $$\sin \delta - \delta \cos \delta >0$$ on (0,π], we observe that $f^{\prime }\left(\delta \right)>0$. This proves that *f* is non-negative on (0,π] and thus proves (ii). □

We continue to prove (v). Setting t=w/δ we have to show that $V(t\delta ) \le t^{d} V(\delta ).$ By (2.1) it suffices to show that for every $1\le t\le \frac{\pi }{\delta }$,

$$\begin{aligned} F(t):= t^{d} \int _0^\delta \sin ^{d-1}s\,\textrm{d}s - \int _0^{t\delta } \sin ^{d-1}s\,\textrm{d}s \ge 0. \end{aligned}$$

Using (i) and (ii) of Claim 2.2 we observe that for every $1\le t\le \frac{\pi }{\delta }$,

$$\begin{aligned} \frac{\partial }{\partial t} \left( \int _0^{t\delta } \sin ^{d-1}s \,\textrm{d}s\right)&= \delta \sin ^{d-1} (t\delta ) \le t^{d-1}\delta \sin ^{d-1}\delta \\&\le t^{d-1} d\int _0^\delta \sin ^{d-1}s \,\textrm{d}s = \frac{\partial }{\partial t}\left( t^{d} \int _0^\delta \sin ^{d-1}s \,\textrm{d}s \right) . \end{aligned}$$

This shows that $F^{\prime }\geq 0$ and implies the desired result as F(1)=0. □

#### Remark 2.3

The asymptotic relation (1.5) for ${{\,\textrm{disp}\,}}_{\mathcal {C}}(d+2,d)$ is an immediate consequence of Lemma 2.1 (iv) and $\arccos x=\frac{\pi }{2}-x-O(x^3)$ as x→0.

If $\frac{\pi }{2}-\varphi$ is of order $\frac{1}{\sqrt{d}}$, then the following lemma gives the asymptotic constant for V(φ) as $d\rightarrow \infty$.

#### Lemma 2.4

For every $$\alpha \ge 0$$,

$$ V\Big (\frac{\pi }{2}-\frac{\alpha }{\sqrt{d}}\Big ) \sim V\Big (\arccos \frac{\alpha }{\sqrt{d+1}}\Big ) \sim \frac{1}{\sqrt{2\pi }}\int _{\alpha }^{\infty }e^{-x^2/2}\,\textrm{d}x\qquad \text {as } \,\, d\rightarrow \infty . $$

#### Proof

The first “∼” follows from $\arccos x=\frac{\pi }{2}-x-O(x^3)$ as x→0. For the second “∼” we interpret $V(\arccos \frac{\alpha }{\sqrt{d+1}})$ as the probability that a uniformly distributed vector on $\mathbb {S}^d$ is contained in a cap of radius $\arccos \frac{\alpha }{\sqrt{d+1}}$ which, without loss of generality, is centered at $e_1=(1,0,\dots ,0)$. Let $g=(g_1,\dots ,g_{d+1})$ be a standard Gaussian vector in $\mathbb {R}^{d+1}$. Then $\frac{g}{\Vert g\Vert _2}$ is uniformly distributed on $\mathbb {S}^d$ and

$$\begin{aligned} \sigma \Big (\Big \{y\in \mathbb {S}^d:\arccos y_1 \le \arccos \frac{\alpha }{\sqrt{d+1}} \Big \}\Big ) =\mathbb {P}\Big [\frac{g_1}{\sqrt{S_{d+1}}}\ge \alpha \Big ], \end{aligned}$$

where $S_{d+1}:=\frac{1}{d+1}\sum _{i=2}^{d+1}g_i^2$. By the law of large numbers $S_{d+1}\rightarrow 1$ in probability and by the continuous mapping theorem the same holds for $\sqrt{S_{d+1}}$. Then Slutsky’s theorem implies that

$$ \mathbb {P}\Big [\frac{g_1}{\sqrt{S_{d+1}}}\ge \alpha \Big ] \rightarrow \mathbb {P}[g_1\ge \alpha ] =\frac{1}{\sqrt{2\pi }}\int _{\alpha }^{\infty }e^{-x^2/2}\,\textrm{d}x. $$

This shows the claimed asymptotic for $V(\arccos \frac{\alpha }{\sqrt{d+1}})$. □

#### Remark 2.5

The asymptotic relation (1.6) for ${{\,\textrm{disp}\,}}_{\mathcal {C}}(2d+2,d)$ directly follows from Lemma 2.4.

We finally provide a standard estimate on the cardinality of geodesic ε-nets on the sphere. Recall that a set of points $\mathcal {N}\subset \mathbb {S}^d$ forms a geodesic ε-net if for every $x\in \mathbb {S}^d$ there is $y\in \mathcal {N}$ such that $\varrho (x,y)\le \varepsilon$, or in other words, the geodesic covering radius defined in the next subsection satisfies $\varphi (\mathcal {N})\le \varepsilon$. Note that for every $x, y\in \mathbb {S}^d$ one has $|x-y|=2\sin (\varrho (x,y)/2)$. The existence of Euclidean or geodesic ε-nets on the sphere $\mathbb {S}^d$ of cardinality $(C/\varepsilon )^d$ for some absolute constant *C* is well-known and follows from the volume argument. We provide a proof with explicit constants for completeness.

#### Lemma 2.6

For every d≥1 and $\varepsilon \in (0,\pi )$ there is a geodesic ε-net $\mathcal {N}\subset \mathbb {S}^d$ of cardinality at most $2(\pi /\varepsilon )^d$.

#### Proof

For d=1 this is straightforward, so let d≥2. If $\varepsilon \ge \frac{\pi }{2}$, then choose $\mathcal {N}=\{x,-x\}$ for some $x\in \mathbb {S}^d$.

For $\varepsilon \in (0,\frac{\pi }{2})$ we use the standard volume argument. Let $\mathcal {N}\subset \mathbb {S}^d$ be a maximal set with $\varrho (x,y)>\varepsilon$ for $x,y\in \mathcal {N}$. Then $\mathcal {N}$ is an ε-net. Since the interiors of balls $B(x,\frac{\varepsilon }{2})$, $x\in \mathcal {N}$, have to be pairwise disjoint, we have

2.4 $$\begin{aligned} 1=\sigma (\mathbb {S}^d)\ge \sum _{x\in \mathcal {N}}\sigma (B(x,\varepsilon /2))=|\mathcal {N}|\, V(\varepsilon /2). \end{aligned}$$

If d=2, then $V(\varepsilon /2)=\frac{1}{2}(1-\cos \frac{\varepsilon }{2})\ge \frac{1}{2}(\frac{\varepsilon }{\pi })^2$, which proves the claim in this case. If d≥3, we use Lemma 2.1 (i) and $\sin \frac{\varepsilon }{2}\ge \frac{\sqrt{2}\varepsilon }{\pi }$, $\varepsilon \in (0, \frac{\pi }{2})$, to obtain that

$$ V(\varepsilon /2) \ge \frac{1}{\sqrt{2\pi (d+1)}}\sin ^d(\varepsilon /2) \ge \sqrt{\frac{2^{d-1}}{\pi (d+1)}}\Big (\frac{\varepsilon }{\pi }\Big )^d \ge \frac{1}{2}\Big (\frac{\varepsilon }{\pi }\Big )^d. $$

Combining this with (2.4) completes the proof. □

### Dispersion and Covering (Proof of Lemma 1.1)

Here we prove Lemma 1.1, which provides relations between minimal dispersion, density, and covering radius, all of which quantify the maximum efficiency of placing points on the sphere. Recall that by definition, given a point set $P=\{x_1,\dots ,x_n\}\subset \mathbb {S}^{d}$, its spherical cap dispersion is

$$ {{\,\textrm{disp}\,}}_{\mathcal {C}}(P) =\sup _{C\in \mathcal {C}}\{\sigma (C):C\cap P=\emptyset \}. $$

With a slight abuse of notations we denote the geodesic covering radius by

$$\begin{aligned} \varphi (P)= &   \inf \Big \{\varphi>0:\bigcup _{i=1}^n B(x_i,\varphi )=\mathbb {S}^d\Big \}\\= &   \sup \{\varphi >0:\exists x\in \mathbb {S}^d \text { with }B(x,\varphi )\cap P=\emptyset \}. \end{aligned}$$

Thus, the geodesic covering radius of *P* equals the geodesic radius of the largest (open) ball which does not intersect *P*. Consequently,

2.5 $$\begin{aligned} {{\,\textrm{disp}\,}}_{\mathcal {C}}(P) =\sup \big \{V(\varphi ):B(x,\varphi )\cap P=\emptyset , x\in \mathbb {S}^d, \varphi \in (0,\pi ) \big \} =V(\varphi (P)). \end{aligned}$$

Taking the infimum over all *n*-point sets $P_n\subset \mathbb {S}^d$ on both sides of this equality and using the continuity of the function V(·) we observe that

2.6 $$\begin{aligned} {{\,\textrm{disp}\,}}_{\mathcal {C}}(n,d) =\inf _{P_n\subset \mathbb {S}^{d}}{{\,\textrm{disp}\,}}_{\mathcal {C}}(P_n) =V\big (\inf _{P_n\subset \mathbb {S}^{d}}\varphi (P_n)\big ) =V(\varphi (n)), \end{aligned}$$

which proves one part of (1.3).

For the relation to minimal density, note that definitions of ${{\,\textrm{dens}\,}}(n,d)$ and φ(n) immediately imply that for every $\varphi >\varphi (n)$ one has ${{\,\textrm{dens}\,}}(n,d)\le n V(\varphi )$ and for every $\varphi <\varphi (n)$ one has ${{\,\textrm{dens}\,}}(n,d)\ge n V(\varphi )$. These two facts together with the continuity of V(·) yield ${{\,\textrm{dens}\,}}(n,d)= n V(\varphi (n))$, which completes the proof. □

### Dispersion and Convex Body Approximation

In this section we present a useful connection between minimal dispersion and best approximation of convex bodies by polytopes. It is well-known that covering $\mathbb {S}^d$ by geodesic balls of equal radius is related to approximating the (d+1)-dimensional Euclidean unit ball $B^{d+1}$ by the convex hull ${{\,\textrm{conv}\,}}(P)$ of the set of centers *P*. Indeed, it is not difficult to see that the largest ball centered at the origin and contained in ${{\,\textrm{conv}\,}}(P)$ has radius $\cos \varphi (P)$, where φ(P) is the geodesic covering radius of *P* defined above (see e.g. [12, Lemma 7.1]). This implies

2.7 $$\begin{aligned} \delta _H({{\,\textrm{conv}\,}}(P),B^{d+1}) =1-\cos \varphi (P), \end{aligned}$$

where the Hausdorff distance between convex sets $K,L\subset \mathbb {R}^{d+1}$ is given by

$$ \delta _H(K,L)=\max \Big \{\sup _{x\in K}\inf _{y\in L}\Vert x-y\Vert ,\,\, \sup _{x\in L}\inf _{y\in K}\Vert x-y\Vert \Big \}. $$

From (2.5) and (2.7) we obtain that, for any *n*-point set $P_n\subset \mathbb {S}^d$,

$$ {{\,\textrm{disp}\,}}_{\mathcal {C}}(P_n) =V(\varphi (P_n)) =f(\delta _H({{\,\textrm{conv}\,}}(P_n),B^{d+1})), $$

with f(x)=V(arccos(1-x)) for $x\in (0,\pi )$. Taking the infimum over all *n*-point sets $P_n\subset \mathbb {S}^d$ on both sides and using continuity of *f* we deduce

2.8 $$\begin{aligned} {{\,\textrm{disp}\,}}_{\mathcal {C}}(n,d) =f(\delta _H(n,d)), \end{aligned}$$

where $\delta _H(n,d)$ denotes the error of best approximation of $B^{d+1}$ (in the Hausdorff distance) by polytopes with at most *n* vertices on $\mathbb {S}^d$.

The relation (2.8) allows to transfer bounds for $\delta _H(n,d)$ to minimal dispersion, and vice versa. This will be used below for the proof of Theorem 1.3. The survey [14] contains many references on the behavior of $\delta _H(n,d)$. Equivalently, one can study the difference $1-\delta _H(n,d)=\cos \varphi (n)$ which is the maximal inradius of a polytope with *n* vertices inscribed into $\mathbb {S}^d$ (see e.g. Lemma 7.1 in [12]) and thus related to Banach-Mazur distance between the Euclidean ball and the convex hull *n* points on the sphere (see discussion in Section 8 of [12]). In [12, inequality (14)] lower bounds for $\cos \varphi (n)$ are given for $d+1\le n\le \sqrt{2}^d$. We will need the following upper bound, which follows from a result on volumes of polytopes with n≥2(d+1) vertices on the sphere, proved independently in [4, 18, 32] and a result on the Banach-Mazur distance between the Euclidean ball and a polytope with $d+2\le n\le 2(d+1)$ vertices, proved in [61]. We formulate it in terms of φ(n) (cf. similar discussion in Section 8 of [12]). It is known that this bound is sharp.

#### Theorem 2.7

Let n≥d+2. Then

$$ \cos \varphi (n) \le C \sqrt{\frac{\ln (n/d)}{d}}, $$

where C>0 is an absolute constant.

## Proofs of Statements

In the following, we provide the proofs of all Theorems and Propositions in order of appearance with four exceptions. Proposition 1.11 and Theorem 1.14 are proved in Section 5, while Propositions 1.12 and 1.13 are proved in Section 4.

### Proof of Theorem 1.3

Recall that $\delta _H(n,d)$ denotes the error of best approximation of $B^{d+1}$ by polytopes with at most *n* vertices on $\mathbb {S}^d$ and that by (2.8),

$$ {{\,\textrm{disp}\,}}_{\mathcal {C}}(n,d)=V(\arccos (1-\delta _H(n,d))). $$

On the other hand it is well known that

3.1 $$\begin{aligned} \delta _{H}(n,d) =\frac{1+o(1)}{2}\Big (\frac{{{\,\textrm{vol}\,}}_d(\mathbb {S}^d)}{{{\,\textrm{vol}\,}}_d(B^d)}\vartheta _{d}\Big )^{2/d}n^{-2/d}\qquad \text {as }n\rightarrow \infty , \end{aligned}$$

where $\vartheta _d$ is the minimal covering density given in (1.7) (see e.g. surveys [36, eq. (4) and (5)] and [14, eq. (7)] and references therein, note that this result goes back to [59]). Combining the asymptotics $\arccos (1-x)=\sqrt{2x}+O(x^{3/2})$ as x→0 and

$$ V(\varphi ) =\frac{d{{\,\textrm{vol}\,}}_d(B^d)}{{{\,\textrm{vol}\,}}_d(\mathbb {S}^d)}\int _0^{\varphi }\sin ^{d-1}t\,\textrm{d}t = (1+o(1))\frac{{{\,\textrm{vol}\,}}_d(B^d)}{{{\,\textrm{vol}\,}}_d(\mathbb {S}^d)}\varphi ^d \qquad \text {as }\varphi \rightarrow 0 $$

with (3.1), we obtain

$$ \lim _{n\rightarrow \infty }n\cdot {{\,\textrm{disp}\,}}_{\mathcal {C}}(n,d) = \vartheta _d \qquad \text {as }n\rightarrow \infty , $$

as claimed. □

### Remark 3.1

Note that in [12, eq. (13)] a slightly less precise asymptotic for $\delta _H(n,d)$ than the one in (3.1) is derived from the covering bound (1.10).

In the next three proofs we will use the following lemma. Recall that V(φ) denotes the normalized Lebesgue measure of a cap of geodesic radius φ and, given a cover $\mathcal {B} =\{B_1, B_2,..., B_m\}$ of the sphere $\mathbb {S}^d$ the density of this cover is $\sum _{i=1}^m \sigma (B_i)$. When the cover $\mathcal {B}$ contains only spherical caps of the same radius φ, we say that $\mathcal {B}$ is a φ-cover and denote its density by ${{\,\textrm{dens}\,}}(\varphi ,\mathcal {B})$. Clearly, ${{\,\textrm{dens}\,}}(\varphi ,\mathcal {B})=m V(\varphi )$.

### Lemma 3.2

Let d≥2, $\varphi _0\in (0, \pi /2]$, and let $D: (0, \varphi _0]\rightarrow [1, \infty )$ be a continuous decreasing function. Assume that for every $\varphi \in (0, \varphi _0]$ there exists a φ-cover $\mathcal {B}$ of the sphere with density ${{\,\textrm{dens}\,}}(\varphi ,\mathcal {B})\le D(\varphi )$. Then for every $n\ge D(\varphi _0)/V(\varphi _0)$ one has $n {{\,\textrm{disp}\,}}_{\mathcal {C}}(n,d)\le D(\varphi (n)).$

### Proof

Let $n\ge D(\varphi _0)/V(\varphi _0)$. Since 1/*V* and *D* are continuous decreasing functions with D≥1 and $V(\varphi )\rightarrow 0$ as $\varphi \rightarrow 0$, there exists a $\psi _n \in (0, \varphi _0]$ such that $n= D(\psi _n)/V(\psi _n)$. By the definition of φ-cover and by the assumptions, there exists $\psi _n$-cover $\mathcal {B}$ of the sphere by *m* spherical caps of radius $\psi _n$ with the density

$$ m V(\psi _n) = {{\,\textrm{dens}\,}}(\psi _n,\mathcal {B}) \le D(\psi _n) = n V(\psi _n). $$

Thus, m≤n so we also have a cover of the sphere by *n* spherical caps of radius $\psi _n$ (just by adding caps if needed). This implies that $\varphi (n)\le \psi _n$. Therefore, using Lemma 1.1, we obtain

$$ n {{\,\textrm{disp}\,}}_{\mathcal {C}}(n,d) = n V(\varphi (n)) \le n V(\psi _n) = D(\psi _n)\le D(\varphi (n)), $$

which completes the proof. □

### Proof of Proposition 1.5

By [12, Theorem 1.1], for every $\varphi \le \frac{\pi }{2}$, there exist centers $x_1,\dots ,x_{m}\in \mathbb {S}^d$ such that every point of $\mathbb {S}^d$ is contained in at least one of the balls in $\mathcal {B}=\{B(x_1,\varphi ),\dots ,B(x_{m},\varphi )\}$ and in at most 400dlnd many. In [12] this is stated for d≥3 but the proof works also for d=2. This implies that for every $\varphi \le \frac{\pi }{2}$

$$\begin{aligned} {{\,\textrm{dens}\,}}(\varphi ,\mathcal {B}) = m\cdot V(\varphi )&=\sum _{i=1}^{m}\int _{\mathbb {S}^d}1_{B(x_i,\varphi )}(y)\,\textrm{d}\sigma (y) \\  &= \int _{\mathbb {S}^d}\sum _{i=1}^{m}1_{B(x_i,\varphi )}(y)\,\textrm{d}\sigma (y) \le 400d\ln d. \end{aligned}$$

Applying Lemma 3.2 with the constant function $D(\varphi )=400d\ln d$ on (0,π/2], we obtain the desired bound for $n\ge \frac{400d\ln d}{V(\pi /2)}=800d\ln d.$ The case n<800dlnd follows from the trivial bounds ${{\,\textrm{disp}\,}}_{\mathcal {C}}(n,d)\le \frac{1}{2}$ (n≥2) and ${{\,\textrm{disp}\,}}_{\mathcal {C}}(1,d)=1$ (cf., (1.4)). □

### Proof of Proposition 1.6

Denote $A_d=d\ln d+d\ln \ln d+5d$. Remark 5.1 and the end of its proof in [12] (see also [48, Theorem 2.2]) imply that for every $\varphi \le \frac{\pi }{2}$ there is a φ-cover $\mathcal {B}$ with ${{\,\textrm{dens}\,}}(\varphi ,\mathcal {B})\le A_d$. Similarly to the proof of Proposition 1.5, using Lemma 3.2 with the constant function $D(\varphi )=A_d$ for $n\ge 2 A_d$ and ${{\,\textrm{disp}\,}}_{\mathcal {C}}(n,d)\le \frac{1}{2}$ for $2\le n< 2 A_d$, ${{\,\textrm{disp}\,}}_{\mathcal {C}}(1,d)=1$ we complete the proof. □

### Proof of Theorem 1.7

Let $\varphi _0:=\arccos \frac{1}{\sqrt{d+1}}$. Corollary 1.2 in [12] states that

$$\begin{aligned} {{\,\textrm{dens}\,}}(\varphi ) \le c d \ln (1+d\cos ^2\varphi ):=D(\varphi ) \end{aligned}$$

on $(0, \varphi _0]$, where c>0 is an absolute constant. Note that $D(\varphi _0)\le c d \ln 2$ and that by Lemma 2.1 (iii),

$$ \frac{1}{3e\sqrt{2\pi }}<V(\varphi _0)<\frac{1}{2}. $$

Applying Lemma 3.2 and Theorem 2.7 we obtain for every $n\ge c_1 d$,

$$ n {{\,\textrm{disp}\,}}_{\mathcal {C}}(n,d)\le c d \ln (1+d\cos ^2(\varphi (n)))\le \frac{c\,d}{n} \ln \Big (1+C^2\ln \frac{n}{d}\Big ) \le \frac{c_2\,d}{n} \ln \ln \frac{n}{d}, $$

where $C, c_1, c_2\ge 2$ are absolute constants. This completes the proof for $n\ge c_1\, d$. For $2d\le n\le c_1\, d$ we use the trivial bound

$$ {{\,\textrm{disp}\,}}_{\mathcal {C}}(n,d) \le 1 \le \frac{C_2\,d}{n} \ln \ln \frac{2n}{d}, $$

for a large enough absolute constant $C_2$. □

### Proof of Corollary 1.8

Let $\varepsilon \in (0,\pi /2)$ and let *n* be the minimal cardinality of a geodesic ε-net. Note that $V(\varepsilon )\le 1/2$. If n≤2d we are done, so we assume m:=n-1≥2d. By the definition of the minimal covering radius we have $V(\varphi (n))\le V(\varepsilon )\le V(\varphi (m))$, therefore Theorem 1.7 and Lemma 1.1 yield

$$ \frac{m}{d}= \frac{m {{\,\textrm{disp}\,}}_{\mathcal {C}}(m,d)}{d\, V(\varphi (m))} \le \frac{C\ln \ln (2m/d)}{V(\varphi (m))} \le \frac{C\ln \ln (2m/d)}{V(\varepsilon )}. $$

This implies

$$ \frac{m}{d}\le C_1\frac{\ln \ln (2/V(\varepsilon ))}{V(\varepsilon )} $$

for a large enough absolute constant $C_1>0$. Since n≤2m, the desired bound follows. □

### Proof of Theorem 1.9

The following proof is based on [12, Example 6.3] where the lower bound by Coxeter-Few-Rogers [21] (the left hand side of (1.8)) is adapted to the sphere. More precisely, in [12, Example 6.3] it was shown that for any finite covering $\mathcal {B}$ of $\mathbb {S}^d$ by caps of equal geodesic radius $\varphi \le \arcsin \sqrt{\frac{1}{d+1}}$ the density satisfies ${{\,\textrm{dens}\,}}(\mathcal {B})\ge c\, d$ with an absolute constant c>0 (although not explicitly stated in [12], the case d=2 is also covered there). Therefore Lemma 1.1 implies that

3.2 $$\begin{aligned} n\cdot V(\varphi (n)) = n\cdot {{\,\textrm{disp}\,}}_{\mathcal {C}}(n,d)={{\,\textrm{dens}\,}}(n,d) \ge c \, d, \end{aligned}$$

whenever $n\ge n_d$ for $n_d$ satisfying $\varphi (n_d)\le \arcsin \frac{1}{\sqrt{d+1}}$, that is,

3.3 $$\begin{aligned} \sin \varphi (n_d)\le \frac{1}{\sqrt{d+1}}. \end{aligned}$$

By the volume bound in Lemma 2.1 (i) and Proposition 1.6 we have

$$ \frac{1}{\sqrt{2\pi (d+1)}}\sin ^d\varphi (n_d) \le V(\varphi (n_d))\le \frac{7 d\ln d}{n_d}. $$

Thus, if $n_d\ge C d^{(3+d)/2}\ln d$ for some suitable absolute constant C>0, then (3.3) holds, which completes the proof. □

## Dispersion Bounds via Geodesic ε-nets

In this section we provide the proofs of Propositions 1.12 and 1.13. For convenience, we mainly use |·| instead of $\sigma (\cdot )$ for the normalized spherical volume and the inverse of the minimal spherical dispersion which is given for $\gamma \in (0,1)$ by

$$ N_{\mathcal {M}}(\gamma ,d):=\min \{n\ge 1 :{{\,\textrm{disp}\,}}_{\mathcal {M}}(n,d)\le \gamma \}, $$

where $\mathcal {M}$ is either $\mathcal {C}$ or $\mathcal {L}$, so we use either $N_{\mathcal {C}}$ or $N_{\mathcal {L}}$ (but in fact it can be any other family of measurable subsets of $\mathbb {S}^d$).

Similarly, for each $\gamma \in (0,1)$ consider

$$\begin{aligned} \mathcal {M}_\gamma := \{ A \in \mathcal {M}: |A| = \gamma \}, \end{aligned}$$

where $\mathcal {M}$ is either $\mathcal {C}$ or $\mathcal {L}$, so we use either $\mathcal {C}_\gamma$ or $\mathcal {L}_\gamma$. As in [2, 45] we call a finite family $\mathcal {N} \subset \mathcal {M}$ a $(c_0 \gamma )$-approximation for $\mathcal {M}_\gamma$ with $c_0 \in (0,1)$, if for every $A \in \mathcal {M}_\gamma$ there exists $B \in \mathcal {N}$ such that B⊂A and $|B| \ge c_0\gamma$. Repeating the proof of [45, Lemma 2.3] (see also [57, Theorem 1]) in our setting we obtain the following.

### Lemma 4.1

Let d≥2 and let $\mathcal {M}$ be either $\mathcal {C}$ or $\mathcal {L}$. Let $c_0\in (0,1)$ and $\gamma \in (0,1)$. If $\mathcal {N}\subset \mathcal {M}$ is a $(c_0\gamma )$-approximation for $\mathcal {M}_{\gamma }$ and if $|\mathcal {N}|\ge 3$, then

$$\begin{aligned} N_{\mathcal {M}}(\gamma , d) \le \frac{3\ln |\mathcal {N}|}{c_0\gamma }. \end{aligned}$$

Moreover, the result holds for the random choice of points.

It is worth mentioning that recently Arman and Litvak in [2, Lemma 3.3] proved a variation of the above result, which again transfers to our setting.

### Lemma 4.2

Let d≥2 and let $\mathcal {M}$ be either $\mathcal {C}$ or $\mathcal {L}$. Let $c_0\in (0,1)$ and $\gamma \in (0,\frac{1}{3c_0})$. If $\mathcal {N}\subset \mathcal {M}$ is a $(c_0\gamma )$-approximation for $\mathcal {M}_{\gamma }$ and if $|\mathcal {N}|\ge \frac{e}{c_0 \gamma }$, then

$$\begin{aligned} N_{\mathcal {M}}(\gamma , d) \le \frac{\ln (4c_0\gamma |\mathcal {N}|)}{c_0\gamma }. \end{aligned}$$

We would like to emphasize that the proof of Lemma 4.1 is based on a random choice of points, while the proof of Lemma 4.2 introduces an additional non-random step.

In order to estimate $N_{\mathcal {M}}(\gamma ,d)$ from above for some $\gamma \in (0,1)$, one can construct a $(c_0\gamma )$-approximation with small cardinality and $c_0=\frac{1}{2}$, say. We provide a quick summary of a construction of such an approximation $\mathcal {N}$ for $\mathcal {M}_\gamma$. Since every cap C(v,δ) from $\mathcal {M}_\gamma$ is uniquely determined by its center *v*, we start with an ε-net $\mathcal {N}_0$ for $\mathbb {S}^d$ and consider $\mathcal {N}$ to be the family of caps centered at points from $\mathcal {N}_0$ with the radius $\delta - \varepsilon$. This guarantees that every cap from $\mathcal {M}_\gamma$ contains a member of $\mathcal {N}$. Moreover, by choosing ε sufficiently small we ensure that their volumes are comparable.

As a tool, we need the following lemma which will be applied first for the family $\mathcal {C}$ and later on for $\mathcal {L}$.

### Lemma 4.3

Let d≥2, $\gamma \in (0,1)$ and $A=B(v_1, \delta _1) \cap B(v_2, \delta _2)$ with some $v_1,v_2\in \mathbb {S}^d$. Let $\delta _1,\delta _2\in (0,\pi ]$ be such that |A|=γ. Then $\delta =\min \{\delta _1,\delta _2\}\ge \gamma$.

### Proof

Assuming $\delta \le \frac{\pi }{2}$ and using the bound in Lemma 2.1 (i) we get

$$ \gamma =|A| \le V(\delta ) \le \frac{\sin ^d \delta }{2} \le \frac{\delta ^d}{2}. $$

Therefore,

$$ \delta \ge \gamma ^{1/d} \ge \gamma . $$

If $\delta >\frac{\pi }{2}$, then $\delta >1\ge \gamma$, which completes the proof. □

To prove Proposition 1.12 we construct a $\frac{\gamma }{2}$-approximation for the family $\mathcal {C}$.

### Lemma 4.4

Let d≥2. For every $\gamma \in (0,1)$ there exists a $\frac{\gamma }{2}$-approximation $\mathcal {N}$ for $\mathcal {C}_{\gamma }$ with cardinality

$$ |\mathcal {N}| \le 2\Big (\frac{3d\pi }{\gamma ^2}\Big )^{d}. $$

### Proof

Let $\gamma \in (0,1)$ and choose $\delta \in (0,\pi )$ such that $V(\delta )=\gamma$. Let $\varepsilon \in (0,\delta )$ and by Lemma 2.6 choose an ε-net $\mathcal {N}_0\subset \mathbb {S}^d$ of cardinality at most $2(\frac{\pi }{\varepsilon })^d$. Then, for every $B(v,\delta )\in \mathcal {C}_{\gamma }$ we find $w\in \mathcal {N}_0$ such that $\varrho (v,w)\le \varepsilon$ and thus $B(w,\delta -\varepsilon )\subset B(v,\delta )$. Let

$$ \mathcal {N}=\{B(w,\delta -\varepsilon ):w\in \mathcal {N}_0\}. $$

Then Lemma 2.1 (v) implies that

$$\begin{aligned} \frac{|B(v,\delta )|}{|B(w,\delta -\varepsilon )|} \le \Big ( \frac{\delta }{\delta -\varepsilon } \Big )^d. \end{aligned}$$

Choosing $\varepsilon = \frac{\gamma \delta }{3 d}$, we observe that

$$\frac{|B(v,\delta )|}{|B(w,\delta -\varepsilon )|} \le \Big ( \frac{1}{1-\frac{\gamma }{3d}} \Big )^d =\Big ( 1+\frac{\gamma }{3d(1-\frac{\gamma }{3d})} \Big )^d \le \Big ( 1+\frac{\gamma }{2d} \Big )^d \le e^{\frac{\gamma }{2}}.$$

This implies

$$\begin{aligned} |B(w,\delta -\varepsilon )| \ge \gamma e^{-\frac{\gamma }{2}}\ge \frac{\gamma }{2}. \end{aligned}$$

Thus the family $\mathcal {N}$ is a $\frac{\gamma }{2}$-approximation for $\mathcal {C}_{\gamma }$ of cardinality at most

$$|\mathcal {N}_0|\le 2\Big (\frac{\pi }{\varepsilon }\Big )^{d} = 2\Big (\frac{3d\pi }{\delta \gamma }\Big )^{d} \le 2\Big (\frac{3d\pi }{\gamma ^2}\Big )^{d}, $$

where we used that $\delta \ge \gamma$ by Lemma 4.3. □

### Proof of Proposition 1.12

Set $\gamma =\frac{12d}{n}\ln n$. If $\gamma \ge 1$, then ${{\,\textrm{disp}\,}}_{\mathcal {C}}(n,d)\le \gamma$ trivially holds and we are done. Assume γ<1. Then, using Lemmas 4.1 and 4.4, we have

$$\begin{aligned}&N_{\mathcal {C}}(\gamma , d) \le \frac{6\ln |\mathcal {N}|}{\gamma } \le \frac{6\ln \Big (2\big (\frac{3\pi d}{\gamma ^2}\big )^{d}\Big )}{\gamma }\\&\quad \le \frac{6d\ln \Big (\frac{5\pi d}{\gamma ^2}\Big )}{\gamma }\le \frac{n}{2}\cdot \frac{\ln (\frac{n^2}{d})}{\ln n} \le n. \end{aligned}$$

This implies ${{\,\textrm{disp}\,}}_{\mathcal {C}}(n,d)\le \gamma =\frac{12d}{n}\ln n$ as required. □

Next we construct an approximation for $\mathcal {L}_\gamma$. The construction that will be used is a natural extension of the one we used for caps in Lemma 4.4, with the only difference that now one has to control not only centers of caps, but also their radii. First we observe that by Lemma 4.3 caps forming an intersection of volume γ have radii from the interval $(\gamma ,\pi ]$. We discretize this interval to approximate radii. Then we construct a finite family of intersections $\mathcal {N}$ using the centers from a given ε-net and radii from the first step which ensures that every lens of volume γ contains a member of the family $\mathcal {N}$. In the final step by choosing ε in an appropriate way we ensure that the volume loss is sufficiently small.

### Lemma 4.5

Let d≥2. For every $\gamma \in (0,1)$ there exists a $\frac{\gamma }{2}$-approximation $\mathcal {N}$ for $\mathcal {L}_\gamma$ with cardinality

$$ |\mathcal {N}| \le 9\Big (\frac{12d\pi }{\gamma ^2}\Big )^{2(d+1)}. $$

### Proof

Fix $\gamma \in (0,1)$. Let $\varepsilon = \frac{\gamma ^2}{12 d}$. By Lemma 2.6 there exists an ε-net $\mathcal {N}_0\subset \mathbb {S}^d$ of cardinality at most $2\big (\frac{\pi }{\varepsilon }\big )^{d}$. By Lemma 4.3, we have $\delta _1,\delta _2\ge \gamma$ and we cover the interval $(\gamma , \pi ]$ by sufficiently small subintervals in the following way:

$$\begin{aligned} (\gamma , \pi ] \subset \bigcup _{i=1}^k \Big (\pi -i\varepsilon , \hspace{0.2cm} \pi - (i-1)\varepsilon \Big ], \end{aligned}$$

where $k \in \mathbb {N}$ is the smallest integer such that $\pi -k\varepsilon \le \gamma$, i.e.,

$$\begin{aligned} k = \Big \lceil \frac{\pi -\gamma }{\varepsilon } \Big \rceil \le \frac{\pi }{\varepsilon } + 1. \end{aligned}$$

Consider now the family

$$\begin{aligned} \mathcal {N}=\{B(w_1, \pi -i\varepsilon ) \cap B(w_2, \pi -j\varepsilon ): w_1,w_2 \in \mathcal {N}_0, i,j=1,2,...,k+1\}. \end{aligned}$$

We show that $\mathcal {N}$ is $\frac{\gamma }{2}$-approximation for $\mathcal {L}_\gamma$ of required cardinality. We first estimate the cardinality. Since $\varepsilon \le \frac{\pi }{4}$,

$$\begin{aligned} |\mathcal {N}_0| \cdot (k+1) \le 2\left( \frac{\pi }{\varepsilon }\right) ^{d} \cdot \left( \frac{\pi }{\varepsilon }+2\right) \le 3\left( \frac{\pi }{\varepsilon }\right) ^{d+1}. \end{aligned}$$

Therefore,

$$\begin{aligned} |\mathcal {N}|= |\mathcal {N}_0\times \mathcal {N}_0| \cdot (k+1)^2 \le 9\left( \frac{\pi }{\varepsilon }\right) ^{2(d+1)} = 9\left( \frac{12\pi d}{\gamma ^2}\right) ^{2(d+1)}. \end{aligned}$$

Next we prove that $\mathcal {N}$ is $\frac{\gamma }{2}$-approximation. Let $A=B(v_1,\delta _1)\cap B(v_2,\delta _2) \in \mathcal {L}_\gamma$. Then i=1,2 there exists $\ell _i \le k$ such that

$$\begin{aligned} \delta _i \in \Big (\pi -\ell _i\varepsilon , \pi - (\ell _i-1)\varepsilon \Big ] \end{aligned}$$

and there exist $w_i \in \mathcal {N}_0$ such that

$$\begin{aligned} B(w_i, \pi -(\ell _i+1)\varepsilon ) \subset B(v_i,\pi -\ell _i\varepsilon )\subset B(v_i,\delta _i). \end{aligned}$$

Since $\delta _i\ge \gamma$ by Lemma 4.3, we have

4.1 $$\begin{aligned} \pi -(\ell _i+1)\varepsilon \ge \delta _i -2\varepsilon =\delta _i - \frac{\gamma ^2}{6 d} \ge \delta _i\Big (1-\frac{\gamma }{6 d}\Big )>0 \end{aligned}$$

for i=1,2. Note that

$$\begin{aligned} B:=B(w_1, \pi -(\ell _1+1)\varepsilon ) \cap B(w_2, \pi -(\ell _2+1)\varepsilon ) \in \mathcal {N} \end{aligned}$$

and B⊂A. Therefore,

4.2 $$\begin{aligned} |B| \ge |A| - |B(v_1, \delta _1) \setminus B(w_1, \pi -(\ell _1+1)\varepsilon )| - |B(v_2, \delta _2) \setminus B(w_2, \pi -(\ell _2+1)\varepsilon )|. \end{aligned}$$

Using Lemma 2.1 (v) and (4.1) we observe

4.3 $$\begin{aligned} \frac{|B(v_i, \delta _i)|}{|B(w_i, \pi -(\ell _i+1)\varepsilon )|} \le \Big ( \frac{1}{1-\frac{\gamma }{6 d}} \Big )^{d} =\Big ( 1+\frac{\gamma }{d(6-\frac{\gamma }{d})} \Big )^{d} \le \Big ( 1+\frac{\gamma }{5d} \Big )^{d} \le e^{\gamma /5}. \end{aligned}$$

Since

$$\begin{aligned} |B(v_i, \delta _i) \setminus B(w_i, \pi -(\ell _i+1)\varepsilon )| = |B(v_i, \delta _i)| - |B(w_i, \pi -(\ell _i+1)\varepsilon )|, \end{aligned}$$

and since $|B(w_i, \pi -(\ell _i+1)\varepsilon )|\le 1$, formula (4.3) implies

$$\begin{aligned} |B(v_i,\delta _i) \setminus B(w_i, \pi -\ell _i\varepsilon )| \le e^{\gamma /5} - 1. \end{aligned}$$

Combining the above estimates with (4.2) and using |A|=γ, and $e^{\gamma /5}\le 1+\frac{\gamma }{4}$ for $\gamma \in (0,1)$, we obtain

$$\begin{aligned} |B| \ge \gamma - 2(e^{\gamma /5}-1) \ge \frac{\gamma }{2}. \end{aligned}$$

As $B\in \mathcal {N}$ and B⊂A, this completes the proof. □

### Proof of Proposition 1.13

We proceed as in the proof of Proposition 1.12. Set $\gamma =\frac{24(d+1)}{n}\ln n$. If $\gamma \ge 1$, then clearly ${{\,\textrm{disp}\,}}_{\mathcal {C}}(n,d)\le 1\le \gamma$. Assume γ<1. By Lemma 4.5, there exists $\frac{\gamma }{2}$-approximation $\mathcal {N}$ for $\mathcal {L}_\gamma$ of cardinality at most $9(\frac{12\pi d}{\gamma ^2})^{2(d+1)}$. Applying this with Lemma 4.1 we obtain

4.4 $$\begin{aligned} N_{\mathcal {L}}(\gamma , d) \le \frac{6\ln \Big (9\big (\frac{12\pi d}{\gamma ^2}\big )^{2(d+1)}\Big )}{\gamma } \le \frac{12(d+1)}{\gamma }\ln \Big (\frac{18\pi d}{\gamma ^2}\Big )\le \frac{n}{2}\cdot \frac{\ln (\frac{n^2}{d})}{\ln n}\le n. \end{aligned}$$

This implies the desired bound. □

### Remark 4.6

In particular, when $\gamma < \frac{1}{d}$ we get

$$N_{\mathcal {L}}(\gamma , d) \le \frac{6(d+1)}{\gamma }\ln (18\pi d) + \frac{12(d+1)}{\gamma }\ln \Big (\frac{1}{\gamma }\Big ) \le \frac{18(d+1)}{\gamma }\ln \Big (\frac{18\pi }{\gamma }\Big ),$$

which is of the same order as the bound given in [51] using the combinatorial ε-net theorem.

### Remark 4.7

By combining Lemma 4.5 with Lemma 4.2 instead of Lemma 4.1 we can improve the absolute constants in (4.4) but at the price of not having random choice of points on the sphere.

## Dispersion Bounds via ε-traversals

In this section we obtain Proposition 1.11 as a consequence of the combinatorial ε-net theorem. To phrase it, let $\mathcal {X}$ be a nonempty set equipped with a σ-algebra Σ. Moreover, let $\mathcal {R}\subset \Sigma$ be a universally separable set system of measurable sets, meaning that there exists a countable subsystem $\mathcal {R}_0\subset \mathcal {R}$ with the following property: *each* $R\in \mathcal {R}$ *can be approximated by a sequence* $R_1,R_2,\dots$ *in* $\mathcal {R}_0$ *in the sense that* x∈R *if and only if* $x\in R_i$ *for all but finitely many indices* *i*. Note that the assumptions are satisfied in the case of $\mathbb {S}^d$ equipped with its Borel σ-algebra and for the choice of $\mathcal {R}=\mathcal {C}$ or $\mathcal {R}=\mathcal {L}_k$ since each cap can be approximated by caps with rational centers and radii.

A suitable notion of dimension in the general framework was given by Vapnik–-Chervonenkis in [65], which is nowadays called the VC-dimension. To introduce it, first define the shatter function of $\mathcal {R}$ by

5.1 $$\begin{aligned} \Pi _{\mathcal {R}}(k)=\max _{\begin{array}{c} P_k\in \mathcal {X}\\ |P_k|=k \end{array}}|\{P_k\cap R:R\in \mathcal {R}\}|, \end{aligned}$$

which returns the maximal number of subsets of a *k*-subset of $\mathcal {X}$ which arise as intersections with $R\in \mathcal {R}$. The VC-dimension of $\mathcal {R}$, given by

$$ {{\,\mathrm{dim_{\textrm{vc}}}\,}}(\mathcal {R})=\sup \{k\in \mathbb {N}_0:\Pi _{\mathcal {R}}(k)=2^k\}, $$

that is, the maximal cardinality of a set such that all its subsets can be realized as intersections with $R\in \mathcal {R}$. The *dispersion* of $P\subset \mathcal {X}$ with respect to $\mathcal {R}$ and a probability measure μ on (X,Σ) is

$$ {{\,\textrm{disp}\,}}(P,\mathcal {R},\mu )=\sup _{R\in \mathcal {R}:R\cap P=\emptyset }\mu (R). $$

The following probabilistic upper bound on the dispersion is essentially contained in [9, Appendix A]. Since other notation, terminology, and concepts are used there, for the reader’s convenience, we present a proof at the end of this section.

### Theorem 5.1

Let μ be a probability measure on (X,Σ). Assume that $d={{\,\mathrm{dim_{\textrm{vc}}}\,}}(\mathcal {R})$ is finite and let m≥d. Suppose that $X_1,\dots ,X_m$ are distributed independently according to μ. Then for all ε>0 one has

$$ \mathbb {P}\Big [{{\,\textrm{disp}\,}}(\{X_1,\dots ,X_m\},\mathcal {R},\mu )>\varepsilon \Big ] \le 2\Big (\frac{2e m}{d}\Big )^{d} 2^{-\varepsilon m/2}. $$

Note that if for a given m≥d we set $\varepsilon =\varepsilon _m=\frac{3}{\ln 2}\frac{d}{m}\ln (\frac{2e m}{d})$, then the probability in Theorem 5.1 becomes strictly less than 1, which leads to existence of an *m*-point set on $\mathcal {X}$ with dispersion at most $\varepsilon _m$. Thus, we only need to have good bounds on the VC-dimension of the class under consideration. For the class of caps it is given by the following lemma.

### Lemma 5.2

Let d≥2. The class $\mathcal {C}=\mathcal {C}_d$ of caps on $\mathbb {S}^d\subset \mathbb {R}^{d+1}$ satisfies ${{\,\mathrm{dim_{\textrm{vc}}}\,}}(\mathcal {C}_d)=d+2$.

For a short proof of Lemma 5.2 we refer to [8, Prop. 5.12] (note that there the notation $\mathbb {S}^n$ is used for (n-1)-dimensional sphere in $\mathbb {R}^n$ and that this fact for d=2 was proved in [1, Prop. 8], where the authors used the definition of VC-dimension which differs by 1 from the standard one). To obtain an upper bound on the dispersion in the case of intersections of caps one can use Lemma 3.2.3 in [9, Lemma 3.2.3], which provides an estimate on the VC-dimension (it implies that the class $\mathcal {L}_k$, consisting of intersections of at most *k* spherical caps, has VC-dimension at most $2 (d+2) k \log _2 (3k)$).

### Proof of Proposition 1.11

Let $X=\mathbb {S}^d$ (equipped with its Borel σ-algebra), $\mathcal {R}=\mathcal {C}$ and $\mu =\sigma$. Since by Lemma 5.2${{\,\mathrm{dim_{\textrm{vc}}}\,}}(\mathcal {C}_d)=d+2$, Theorem 5.1 yields that for every m≥d+2 there exists an *m*-point set $P_m\subset \mathbb {S}^d$ with

$$\begin{aligned} {{\,\textrm{disp}\,}}_{\mathcal {C}}(P_m)\le \varepsilon _m:=\frac{3}{\ln 2}\, \frac{d+2}{m}\, \ln \Big (\frac{2e m}{d+2}\Big ). \end{aligned}$$

In particular, this gives ${{\,\textrm{disp}\,}}_{\mathcal {C}}(m,d)\le \varepsilon _m$ and completes the proof.

### Proof of Theorem 1.14 and inequality (1.11)

We repeat the proof above (and essentially follow [51]). Using that ${{\,\mathrm{dim_{\textrm{vc}}}\,}}(\mathcal {C}_d)=d+2$ and [9, Lemma 3.2.3], we observe that ${{\,\mathrm{dim_{\textrm{vc}}}\,}}(\mathcal {L}_k)\le 2 (d+2) k \log _2 (3k)$, in particular, ${{\,\mathrm{dim_{\textrm{vc}}}\,}}(\mathcal {L}_2)={{\,\mathrm{dim_{\textrm{vc}}}\,}}(\mathcal {L})\le 11(d+2)$. Therefore Theorem 5.1 implies

$$ {{\,\textrm{disp}\,}}_{\mathcal {L}_k}(m,d) \le \frac{6}{\ln 2}\, \frac{(d+2) k \log _2 (3k)}{m}\, \ln \Big (\frac{e m}{ (d+2) k \log _2 (3k)}\Big ). $$

for every $m\ge 2 (d+2) k \log _2 (3k)$ (in the case of $\mathcal {L}$, that is, in the case k=2, it is enough to ask m≥11(d+2)). □

### Remark 5.3

Note that Theorem 5.1 yields the existence of an absolute constant C>1 such that for every $\varepsilon \in (0,\frac{1}{2})$ there exists a point set $P_n\subset \mathcal {X}$ of cardinality

$$\begin{aligned} n\le \frac{C{{\,\mathrm{dim_{\textrm{vc}}}\,}}(\mathcal {R})}{\varepsilon }\ln \frac{1}{\varepsilon } \end{aligned}$$

with ${{\,\textrm{disp}\,}}(P_n,\mathcal {R},\mu )<\varepsilon$. It was shown in [44, Theorem 2.1] that in general this bound on *n* is sharp up to an absolute constant and in [44, Theorem 3.1] that for sufficiently small ε>0 (depending on ${{\,\mathrm{dim_{\textrm{vc}}}\,}}(\mathcal {R})$) one may take *C* arbitrarily close to 1. Note however that for the proof of Proposition 1.11 we require bounds valid for all $\varepsilon \in (0,\frac{1}{2})$.

### Remark 5.4

The notion of VC-dimension can also be used to bound the size of empirical processes or (spherical cap) discrepancy, see [38, 60].

The remainder of this section is devoted to the proof of Theorem 5.1, which is essentially taken from [9, Appendix A] and [37].

A set $P\subset \mathcal {X}$ is an ε*-traversal* of $\mathcal {R}$ if $R\cap P\ne \emptyset$ for all

$$ R\in \mathcal {R}_{\varepsilon }:=\{R\in \mathcal {R}:\mu (R)> \varepsilon \}. $$

This definition is more general than the one of (combinatorial) ε-nets introduced in [37]. Theorem 5.1 provides an upper bound on the minimal cardinality of ε-traversals and thus generalizes the corresponding result for ε-nets in [37].

Given ε>0 and m≥1 consider the set

$$ Q^m_{\varepsilon } =\{(x_1,\dots ,x_m)\in \mathcal {X}^m:\exists R\in \mathcal {R}_{\varepsilon }\,\, \text{ with } \,\, R\cap \{x_1,\dots ,x_m\}=\emptyset \} $$

of realizations of $\{X_1,\dots ,X_m\}$ not forming an ε-traversal. Then the probability that $\{X_1,\dots ,X_m\}$ has dispersion >ε is given by $\mu ^{\otimes m}(Q^m_{\varepsilon })$, where $\mu ^{\otimes m}$ denotes the product measure on $X^m$. Also define the set

$$\begin{aligned} J^{2m}_{\varepsilon }=\Big \{&(\bar{x},\bar{y})=(x_1,\dots ,x_m,y_1,\dots ,y_m)\in \mathcal {X}^{2m}:\\&\exists R\in \mathcal {R}_{\varepsilon }\, \text{ such } \text{ that } \, R\cap \{x_1,\dots ,x_m\}=\emptyset \, \text{ and } \, |R\cap \{y_1,\dots ,y_m\}|\ge \frac{\varepsilon m}{2}\Big \}. \end{aligned}$$

### Lemma 5.5

Let ε>0 and m≥1 be such that $m\varepsilon \ge 2$. Then

$$ \mu ^{\otimes 2m}(J^{2m}_{\varepsilon }) \le \mu ^{\otimes m}(Q^m_{\varepsilon }) \le 2 \mu ^{\otimes 2m}(J^{2m}_{\varepsilon }). $$

### Proof

If $\varepsilon \ge 1$, then $\mathcal {R}_{\varepsilon }=\emptyset$ and the statement is trivial. Thus assume $\varepsilon \in (0,1)$. The first inequality follows since $J^{2m}_{\varepsilon }$ contains a stronger condition and by Fubini’s theorem.

To prove the second bound, note that by Fubini’s theorem,

$$\begin{aligned} \mu ^{\otimes 2m}(J^{2m}_{\varepsilon })&=\int _{\mathcal {X}^m}\int _{\mathcal {X}^m}\textbf{1}_{J^{2m}_{\varepsilon }}(\bar{x},\bar{y})\,\textrm{d}\mu ^{\otimes m}(\bar{y})\,\textrm{d}\mu ^{\otimes m}(\bar{x})\\&\ge \int _{Q^m_{\varepsilon }}\int _{\mathcal {X}^m}\textbf{1}_{J^{2m}_{\varepsilon }}(\bar{x},\bar{y})\,\textrm{d}\mu ^{\otimes m}(\bar{y})\,\textrm{d}\mu ^{\otimes m}(\bar{x}). \end{aligned}$$

We estimate the inner integral uniformly from below. Fix $\bar{x}\in Q^m_{\varepsilon }$. Then there exists $R\in \mathcal {R}_{\varepsilon }$ such that $R\cap \{x_1,\dots ,x_m\}=\emptyset$. We fix one such *R* and denote it by $R_{\bar{x}}$. Let $K^{2m}_{\varepsilon }=K^{2m}_{\varepsilon }(\bar{x})$ be the set of $(\bar{x},\bar{y})\in \mathcal {X}^{2m}$ with $|\{y_1,\dots ,y_m\}\cap R_{\bar{x}}|\ge \varepsilon m/2$. Then $K^{2m}_{\varepsilon }\subset J^{2m}_{\varepsilon }$ and hence

$$\begin{aligned} \int _{\mathcal {X}^m}\textbf{1}_{J^{2m}_{\varepsilon }}(\bar{x},\bar{y})\,\textrm{d}\mu ^{\otimes m}(\bar{y})&\ge \int _{\mathcal {X}^m}\textbf{1}_{K^{2m}_{\varepsilon }}(\bar{x},\bar{y})\,\textrm{d}\mu ^{\otimes m}(\bar{y})\\&= \int _{\mathcal {X}^m}\textbf{1}_{\{|\{y_1,\dots ,y_m\}\cap R_{\bar{x}}|\ge \varepsilon m/2\}}(\bar{y})\,\textrm{d}\mu ^{\otimes m}(\bar{y}). \end{aligned}$$

The latter integral is identical to the probability that $\sum _{i=1}^{m}\xi _i\ge \varepsilon m/2$, where $\xi _i=\textbf{1}_{Y_i\in R_{\bar{x}}}$ and $Y_1,\dots ,Y_m$ are independently distributed according to μ. Then the random variables $\xi _1,\dots ,\xi _m$ are independent Bernoulli random variables distributed with paramater $p=\mu (R_{\bar{x}})>\varepsilon$. We set p=ε which only decreases the above probability. Since any median *M* of the Binomial distribution with parameters m,ε satisfies $M\ge m\varepsilon -1$, we observe that for $m\ge 2/\varepsilon$,

$$ \mathbb {P}\Big [\sum _{i=1}^{m}\xi _i\ge \varepsilon m/2\Big ] \ge \frac{1}{2}. $$

Therefore,

$$ \mu ^{\otimes 2m}(J^{2m}_{\varepsilon }) \ge \frac{1}{2}\int _{Q^m_{\varepsilon }}\,\textrm{d}\mu ^{\otimes m}(\bar{x}) =\frac{1}{2}\mu (Q^m_{\varepsilon }), $$

which proves the lemma. □

We need another lemma. Recall that $\Pi _{\mathcal {R}}$ was defined in (5.1).

### Lemma 5.6

For all ε>0 and m≥1,

$$\begin{aligned} \mu ^{\otimes 2m}(J^{2m}_{\varepsilon })\le \Pi _{\mathcal {R}} (2m) \, 2^{-\varepsilon m/2}. \end{aligned}$$

### Proof

A permutation π of $\{1,\dots ,2m\}$ acts on $\bar{z}=(z_1,\dots ,z_{2m})\in \mathcal {X}^{2m}$ by $\pi (\bar{z})=(z_{\pi (1)},\dots ,z_{\pi (2m)})$ and satisfies $\pi \circ \mu ^{\otimes 2m}=\mu ^{\otimes 2m}$. Averaging over all permutations yields

$$\begin{aligned} \mu ^{\otimes 2m}(J^{2m}_{\varepsilon })&=\int _{\mathcal {X}^{2m}}\textbf{1}_{J^{2m}_{\varepsilon }}(\bar{z})\,\textrm{d}\mu ^{\otimes 2m}(\bar{z})\\&=\int _{\mathcal {X}^{2m}}\Big (\frac{1}{(2m)!}\sum _{\pi }\textbf{1}_{J^{2m}_{\varepsilon }}(\pi (\bar{z}))\Big )\,\textrm{d}\mu ^{\otimes 2m}(\bar{z}), \end{aligned}$$

where the sum is taken over all (2*m*)! such permutations. We provide a uniform upper bound on the integrand.

Fix $\bar{z}=(z_1,\dots ,z_{2m})\in \mathcal {X}^{2m}$ and let $Z=\{z_1,\dots ,z_{2m}\}$. Recall that $\pi (\bar{z})\in J_{\varepsilon }^{2m}$ if and only if there is $R\in \mathcal {R}_{\varepsilon }$ such that $R\cap \{z_{\pi (1)},\dots ,z_{\pi (m)}\}=\emptyset$ and $|R\cap \{z_{\pi (m+1)},\dots ,z_{\pi (2m)}\}|\ge \frac{\varepsilon m}{2}$. In this case we write $\pi \leftrightarrow R$. Without loss of generality, we replace $\mathcal {R}_{\varepsilon }$ by the smaller set system

$$\begin{aligned} \mathcal {R}_{\varepsilon }|_{\bar{z}}=\{R\cap \{z_{1},\dots ,z_{2m}\}:R\in \mathcal {R}_{\varepsilon }\}. \end{aligned}$$

Given $R\in \mathcal {R}_{\varepsilon }|_{\bar{z}}$ define the set of permutations $S_R=\{\pi :\pi \leftrightarrow R\}$. Then $\pi (\bar{z})\in J_{\varepsilon }^{2m}$ if and only if $\pi \in S_R$ for some (possibly non-unique) $R\in \mathcal {R}_{\varepsilon }|_{\bar{z}}$. This gives

$$ \frac{1}{(2m)!}\sum _{\pi }\textbf{1}_{J^{2m}_{\varepsilon }}(\pi (\bar{z})) \le \frac{1}{(2m)!}\sum _{\pi }\sum _{R\in \mathcal {R}_{\varepsilon }|_{\bar{z}}}\textbf{1}_{\pi \in S_R} =\sum _{R\in \mathcal {R}_{\varepsilon }|_{\bar{z}}}\frac{1}{(2m)!}\sum _{\pi } \textbf{1}_{\pi \in S_R}. $$

Let $R\in \mathcal {R}_{\varepsilon }|_{\bar{z}}$. If $S_R=\emptyset$, omit *R* from the first sum. If $S_R\ne \emptyset$, then there exists a subset of distinct indices $I_R=\{i_1,\dots ,i_{\ell }\}$ such that $\frac{\varepsilon m}{2}\le \ell \le m$ and $\{z_1,\dots ,z_{2m}\}\cap R=\{z_{i_1},\dots ,z_{i_{\ell }}\}$ and $z_i\not \in R$ for $i\not \in I_R$. Note that for a permutation π, $\pi \in S_R$ if and only if $\pi ^{-1}(i_1),\dots ,\pi ^{-1}(i_{\ell })\in \{m+1,\dots ,2m\}$. The fraction of permutations satisfying this can be computed by noting that there are $$\left( {\begin{array}{c}m\\ \ell \end{array}}\right) $$ ways to map $I_R$ into $\{m+1,\dots ,2m\}$ but $$\left( {\begin{array}{c}2m\\ \ell \end{array}}\right) $$ ways to map $I_R$ into $\{1,\dots ,2m\}$. Therefore,

$$ \frac{1}{(2m)!}\sum _{\pi } \textbf{1}_{\pi \in S_R} =\frac{\left( {\begin{array}{c}m\\ \ell \end{array}}\right) }{\left( {\begin{array}{c}2m\\ \ell \end{array}}\right) } =\frac{m(m-1)\cdots (m-\ell +1)}{2m(2m-1)\cdots (2m-\ell +1)} \le 2^{-\ell } \le 2^{-\varepsilon m/2}. $$

We complete the proof by noting that

$$ |R_{\varepsilon }|_{\bar{z}}|\le |\{R\cap \{z_1,\dots ,z_{2m}\}:R\in \mathcal {R}\}|\le \Pi _{\mathcal {R}}(2m). $$

□

These two lemmas yield that for every ε>0 and m≥1 with $m\varepsilon \ge 2$,

$$ \mu ^{\otimes m}(Q^m_{\varepsilon }) \le 2\mu ^{\otimes 2m}(J^{2m}_{\varepsilon }) \le 2\Pi _{\mathcal {R}}(2m)2^{-\varepsilon m/2}. $$

It remains to bound the shatter function in terms of the VC-dimension. It is done by the Sauer-Shelah lemma, which goes back to independent works by Vapnik and Chervonenkis, by Sauer, and by Shelah, see the references in [47] related to Lemma 5.9 there.

### Lemma 5.7

Let $\mathcal {R}\subset \Sigma$ be a set system with ${{\,\mathrm{dim_{\textrm{vc}}}\,}}(\mathcal {R})\le d$. Then for m≥1,

$$ \Pi _{\mathcal {R}}(m)\le \Phi _d(m):=\sum _{k=0}^{d}\left( {\begin{array}{c}m\\ k\end{array}}\right) . $$

### Proof of Theorem 5.1

Let m≥d and ε>0. If mε<2, then the bound on the probability is trivially satisfied. Thus, let $m\varepsilon \ge 2$. We bound the sum in Lemma 5.7 using the standard bound

$$ \sum _{k=0}^{d}\left( {\begin{array}{c}m\\ k\end{array}}\right) \le \Big (\frac{me}{d}\Big )^{d}, $$

which follows from

$$ e^d \ge \Big (1+\frac{d}{m}\Big )^m \ge \sum _{k=0}^{d}\Big (\frac{d}{m}\Big )^{k}\left( {\begin{array}{c}m\\ k\end{array}}\right) \ge \Big (\frac{d}{m}\Big )^{d}\sum _{k=0}^{d}\left( {\begin{array}{c}m\\ k\end{array}}\right) . $$

Combined with Lemmas 5.5, 5.6 and 5.7, we obtain

$$ \mu ^{\otimes m}(Q^m_{\varepsilon }) \le 2\Big (\frac{2 e m}{d}\Big )^{d}2^{-\varepsilon m/2}. $$

This completes the proof. □

## Data Availability

We do not analyse or generate any datasets, because our work proceeds within a theoretical approach.
